# Distinct Changes in Synaptic Protein Composition at Neuromuscular Junctions of Extraocular Muscles versus Limb Muscles of ALS Donors

**DOI:** 10.1371/journal.pone.0057473

**Published:** 2013-02-26

**Authors:** Jing-Xia Liu, Thomas Brännström, Peter M. Andersen, Fatima Pedrosa-Domellöf

**Affiliations:** 1 Department of Integrative Medical Biology, Section for Anatomy, Umeå University, Umeå, Sweden; 2 Department of Medical Biosciences, Pathology, Umeå University, Umeå, Sweden; 3 Department of Pharmacology and Clinical Neuroscience, Umeå University, Umeå, Sweden; 4 Department of Clinical Sciences, Ophthalmology, Umeå University, Umeå, Sweden; University of Rome La Sapienza, Italy

## Abstract

The pathophysiology of amyotrophic lateral sclerosis (ALS) is very complex and still rather elusive but in recent years evidence of early involvement of the neuromuscular junctions (NMJs) has accumulated. We have recently reported that the human extraocular muscles (EOMs) are far less affected than limb muscles at the end-stage of ALS from the same donor. The present study aimed to compare the differences in synaptic protein composition at NMJ and in nerve fibers between EOM and limb muscles from ALS donors and controls. Neurofilament light subunit and synaptophysin decreased significantly at NMJs and in nerve fibers in limb muscles with ALS whereas they were maintained in ALS EOMs. S100B was significantly decreased at NMJs and in nerve fibers in both EOMs and limb muscles of ALS donors, but other markers confirmed the presence of terminal Schwann cells in these NMJs. p75 neurotrophin receptor was present in nerve fibers but absent at NMJs in ALS limb muscles. The EOMs were able to maintain the integrity of their NMJs to a very large extent until the end-stage of ALS, in contrast to the limb muscles. Changes in Ca^2+^ homeostasis, reflected by altered S100B distribution, might be involved in the breakdown of nerve-muscle contact at NMJs in ALS.

## Introduction

Amyotrophic lateral sclerosis (ALS) is an adult-onset neurodegenerative disorder affecting both upper and lower motor neurons and resulting in skeletal muscle weakness and atrophy [1]. The pathophysiology of ALS is very complex and still rather elusive but in recent years evidence of early involvement of the neuromuscular junctions (NMJs) has accumulated [2]. Signs of retrograde axonal degeneration detected by MRI have been reported in ALS patients [3], [4] and retraction of motor axons from their muscle synapse has been shown to occur before any symptoms of the disease appear in the limb muscles of transgenic SOD1^G93A^ mice, the most widely used ALS rodent model [5]. We have recently reported that the human extraocular muscles (EOMs) are far less affected at the end-stage of ALS than limb muscles from the same donor [6], [7]. We have also shown that loss of motor axon contact at the NMJs does not occur in the EOMs of the SOD1^G93A^ mice [8]. However, the “end-stage” of the disease in the SOD1^G93A^ mice is set to the point when the animals no longer can feed themselves due to the ethical limitations of animal experiments. In contrast, in patients with ALS, the disease progresses far beyond that stage, even in the absence of assisted ventilation. Furthermore, we have previously shown that there are distinct changes in the distribution of laminin chain isoforms in the NMJs of EOM *vs* limb muscles of ALS donors [7]. The most striking difference was with regard to laminin α4-chain which had disappeared from the majority of NMJs of limb muscles but was still present in those of the EOMs. Differences in laminin composition of the basal laminin of the nerves were also apparent between the EOMs and limb muscles of ALS donors [7]. In summary, in the context of motor axon retraction being part of the pathogenesis of ALS, it is of particular interest to further investigate and compare the changes in the cellular and molecular microenvironment at NMJs in EOM and limb muscles of human donors.

At the peripheral NMJ, three different cell types, i.e. motor nerve terminal, terminal Schwann cells and muscle fibers, collaborate in the assembly and maintenance of the synaptic apparatus. The NMJ is highly specialized and has remarkable plasticity, changing its shape, size and molecular organization as it undergoes pathological processes [9], [10]. In the present study, we used markers of the different elements of the NMJ: neurofilament (NF) and synaptophysin to study the nerve endings, S100B, p75 neurotrophin receptor (p75^NTR^) and glial fibrillary acidic protein (GFAP) to study the terminal Schwann cells, and α-bungarotoxin to detect the muscle side of the synapse, in order to further elucidate the differences in the impact of ALS on EOM *vs* limb NMJs. NF proteins are present in nerve axons and dendrites and comprise three subunits (70, 160, and 200-kD) [11]. Together with other neuronal cytoskeleton proteins, actin microfilaments and microtubules, NFs provide structural support to the nerve cells [12]. Synaptic vesicles, which are the crucial organelles in neurotransmitter release, accumulate at sites of nerve-muscle contact and they contain different functional types of synaptic vesicle proteins. Synaptophysin is a 38 kD integral membrane glycoprotein of synaptic vesicles that is concentrated at active zones in the presynaptic region at NMJs and is therefore regarded as a good marker of functioning NMJs [13], [14]. S100B, a well-known Schwann cell marker, is an acidic calcium-binding protein with a molecular weight of 21 kDa, originally isolated from brain tissue [15] and consisting of two subunits, S100A and S100B. S100B is also known as astrocyte-derived neurothrophic protein since it is mainly localized in glial cells in the central nervous system and in Schwann cells in the peripheral nervous system [16]. It has been reported that S100B protein decreases in serum [17] and cerebrospinal fluid [18] but increases in spinal cord motor neurons [19] of ALS patients. GFAP and p75^NTR^ are also widely used as standard Schwann cell markers. GFAP is a glial intermediate filament component and generally considered as a late marker for Schwann cells since it appears at a relatively late stage of Schwann cell development and it is down-regulated in those Schwann cells which form myelin [20], [21]. Up-regulation of GFAP occurs in Schwann cells after nerve injury [21]. Terminal Schwann cells are nonmyelinating Schwann cells and are strongly labeled by antibodies against GFAP [22]. p75^NTR^ is a member of the tumor necrosis factor receptor family and binds the members of the neurotrophin family including nerve growth factor, brain-derived neurotrophin factor, neurotrophin-3, neurotrophin-4 and even pro-forms of nerve growth factor and neurotrophins [23]. p75^NTR^ expression is temporally regulated showing high levels during development whereas it is down-regulated significantly after birth. However, in the event of peripheral nerve injury, p75^NTR^ can be re-expressed to a considerable degree [24], [25].

The human EOMs have a distinct gene expression profile [26] and their response to neuromuscular disease differs significantly from that of limb muscles. Because the EOMs are remarkably preserved compared to limb muscles in terminal ALS, although they are not completely unaffected, a better understanding of the distinct response of these muscles to ALS may provide useful clues to delay the progress of the disease.

## Materials and Methods

### Ethics Statements

All human muscle samples were collected at autopsy with the approval of the Ethical Committee of Umeå University and the Regional Ethical Review Board in Umeå, section for Medical Research, adhering to the principles of the Declaration of Helsinki. Information about the study was given orally and in written format to next of kin, and in most cases also to the patient. Informed consent was obtained from next of kin from all patients prospectively enrolled in the study, the oral consent was then noted in the hospital files. The use of retrospective archival material from the hospital laboratory of clinical pathology was also approved. The animal study has been conducted according to national and international guidelines. Experiments and animal handling were approved by the Ethical Committee of the Medical Faculty, Umeå University and were carried out in accordance with the European Communities’ Council Directive (86/609/EEC).

### Human Subjects

A total of 23 EOM and 10 limb muscle samples were collected from seven ALS donors. Detailed information about sex, age, and major clinical features of ALS donors is given in Table 1. Normal EOM samples from ten control subjects (age 34, 34, 42, 47, 53 yr, here referred to as “adult”; age 65, 71, 72, 73 and 87 yr, here referred to as “elderly”) were collected at autopsy. Normal limb muscle samples from 5 adult (age 23, 24, 34, 55, and 58 yr) and 5 elderly (age 69, 76, 78, 81 and 83 yr) controls were also collected at autopsy. None of the control subjects was known to suffer from neuromuscular disease. Muscle samples were mounted, rapidly frozen in propane chilled with liquid nitrogen and stored at –80 °C until processed. Serial cross-sections (7 µm thick for conventional fluorescence microscopy and 40 µm thick for confocal microscopy) were cut across the whole EOM or the whole limb muscle samples in a cryostat (Reichert Jung; Leica, Nussloch, Germany).

**Table 1:** Characteristics of ALS donors and extraocular (EOM) and limb muscles examined in each donor.

Donor	Sex	Age atdeath (yr)	Symptom duration (months)	Diagnosis	SOD1 genotype	Site of 1^st^ symptom	EOM	Limb
Da*	Male	78	84	FALS	D90A/D90A	bulbar onset	–	VL
D1	Male	80	31	SALS	wt/wt	right hand	RS, OS, RL(2)	BB, VL
D2	Male	75	317	FALS	D90A/D90A	left leg	RL (3), RM (2)	TA
D3	Female	64	132	FALS	D90A/D90A	left leg	RL	BB (2)
D4	Female	80	12	SALS	wt/wt	bulbar onset	RL (3), RM, RS	VL
D5	Male	66	13	SALS	wt/wt	bullar onset	OS (2)	BB
D7	Female	58	50	SALS	wt/wt	bulbar onset	RS (2), OS, RL, RM (2)	VL (2)

* The donors were numbered according to the order in which they were collected for EOM vs limb studies. For consistency with the previous donor numbers [6], the newly added and earliest donor of limb muscle only was named Da.

RS, rectus superior; RL, rectus lateralis; RM, rectus medialis; OS, obliquus superior; VL, vastus lateralis; BB, biceps brachii; TA, tibialis anterior. The total number of samples studied is given in brackets. wt = wild type phenotype.

### Mouse Samples

The EOMs and hind limb muscles from SOD1^G93A^ mice [27] at terminal stage (n = 4; 142–165 days) and age-matched C57BL/6 controls (n = 4; 128–167 days) were collected directly after the animals were sacrificed with an intraperitoneal injection of pentobarbital, and processed as above.

### Antibodies and Labeling

NMJs were visualized using either fluorescent-conjugated α-bungarotoxin (α-BTx) (Molecular Probes Inc., Eugene, OR, USA) or a monoclonal antibody against acetylcholine receptor alpha subunit (AbD Serotec, Raleigh, NC, USA), which both bind to post-synaptic acetylcholine receptors on the plasma membrane of muscle fibers. The following monoclonal antibodies were used: anti-neurofilament 70 kD (clone NR4; DAKO; Glostrup, Denmark; here referred to as NF-L to denote that it only recognizes the light NF subunit) used for the detection of cells of neuronal origin; anti-synaptophysin (clone SY 38; Boehringer Mannheim Biochemica, Indianapolis, IN, USA) against presynaptic vesicles of neuromuscular endplates; anti-S100B (S2532, Sigma-Aldrich, St. Louis, MO, USA) used as a Schwann cell marker. Polyclonal antibodies against GFAP (Z0334, DAKO, Glostrup, Denmark) and p75^NTR^ (G3231, Promega, Madison, WI, USA) were also used as Schwann cell markers. Polyclonal anti-S100 antibody against human S100A and B (S100; Z0311, DAKO; Glostrup, Denmark) was used on mouse samples. In addition, monoclonal antibody 2B6 (gift from Dr. Neal Rubinstein, University of Pennsylvania, Philadelphia, USA) against embryonic myosin heavy chain isoform was used to identify regenerating muscle fibers [28]. Immunohistochemistry was performed on air-dried serial consecutive tissue sections rehydrated in 0.01M PBS, and then immersed in 5% normal goat serum (Dakopatts; Glostrup, Denmark) for 15 min for 7 µm sections and 30 min for 40 µm sections. Sections were then incubated with the appropriate primary antibody at +4°C overnight. All antibodies were diluted in 0.01M PBS containing 0.1% bovine serum albumin and used at their optimal dilutions. After washing, sections were further incubated for 1 hr (for 7 µm sections) or 1.5 hrs (for 40 µm sections) at 37°C with a mixture of goat anti-rabbit secondary antibody (Alexa 488 for green fluorescence; Molecular Probes Inc., Eugene, OR, USA) and goat anti-mouse secondary antibody (Alexa 594 for red fluorescence; Molecular Probes Inc., Eugene, OR, USA) or a mixture of goat anti-mouse secondary antibody (Alexa 488 for green fluorescence; Molecular Probes Inc., Eugene, OR, USA) plus rhodamine-conjugated α-BTx. Control sections were treated as above, except that the primary antibody was either omitted or substituted by non-immune serum.

### Quantification of NMJs

For each muscle sample, double-stained sections combining α-BTx or AChR antibody and each of the antibodies to be studied (α-BTx+NF-L, α-BTx+synapatophysin, α-BTx+S100B, AChR+GFAP, AChR+p75^NTR^, for human and α-BTx+S100 for mouse, on separate sections) were evaluated. The total area of each muscle section was examined and each NMJ identified with α-BTx/AChR was counted and evaluated as either “labeled” or “unlabeled” with the antibody used. The total number of α-BTx/AChR-positive NMJs was recorded as well as the total number of NMJs that were labeled with the specific antibody. Care was taken to make sure that i) the α-BTx staining was true and on the surface of a muscle fiber; ii) the antibody labeling was truly higher than background level; iii) auto-fluorescence was excluded; iv) no NMJs were counted more than once; v) partial occupancy and particular aspects of NMJ morphology (en grappe/en plaque motor end-plates) were taken into consideration. The 7 µm sections were examined using a Nikon microscope (Eclipse, E800; Melville, NY, USA) equipped with a Spot RT colour camera (Dignostic Instruments Inc., MI, USA). Computer generated images were processed using the Adobe Photoshop software (Adobe System Inc., Mountain View, CA, USA). The 40 µm sections were examined using a Leica TSP-2 confocal microscopy (Heidelberg, Germany) and the images were analyzed using Leica LCS software (Leica, Heidelberg, Germany).

### Statistical Analysis

The percentage of labeled NMJs was calculated for each muscle sample and each antibody (Table 2, Table 3). Means and standard errors and unpaired *t*-test were calculated and statistics analysis was performed with the help of StatView software (SAS Institution Inc., Cary, NY). Statistically significant difference was considered at *p*<0.05.

**Table 2:** Percentage of NMJs labeled with anti-NF-L, -synaptophysin, -S100B, -GFAP or -p75^NTR^ in EOMs of ALS and controls.

Donors	NF-L	SYP	S100B	GFAP	p75^NTR^
D1	76% (N = 21)	82% (N = 11)	37% (N = 57) (6%–69%)^a^	100% (N = 41)	70% (N = 40)
D2	76% (64%–87%) (N = 215)	84% (74%–91%) (N = 79)	10% (N = 74)	89% (N = 52)	83% (N = 23)
D4	88% (67)	91% (N = 85) (86%–95%)	30% (N = 74) (27%–34%)	95% (N = 49) (91%–100%)	93% (N = 59) (93%–94%)
D5	90% (N = 19)	88% (N = 32)	20% (N = 50)	91% (N = 44)	89% (N = 35)
D7	69% (N = 13)	80% (N = 20)	19% (N = 171) (6%–43%)	93% (N = 112) (83%–100%)	89% (N = 117) (84%–92%)
Mean±SE	77%±3.5 (N = 357)	85%±2.3 (N = 380)	25%±7.1* (N = 426)	94%±2.4 (N = 298)	87%±2.8 (N = 274)
**Controls**					
Adult (n = 5)	82%±5.4 (N = 206)	88%±1.5 (N = 260)	78%±11.8 (N = 264)	94%±2.5 (N = 131)	92%±5.6 (N = 127)
Elderly (n = 5)	80%±4.0 (N = 92)	84%±2.9 (N = 143)	82%±3.9 (N = 66)	100%±0 (N = 40)	87%±13.3 (N = 45)

a The range of the percentage of the labeled NMJs is given when more than one muscle specimen from the same donor was examined.

* Statistically significant difference between ALS donors and elderly (p = 0.005), and adult (p = 0.01) controls.

N: the number of NMJs examined; n: the number of control subjects studied.

**Table 3:** Percentage of NMJs labeled with anti-NF-L, -synaptophysin, -S100B, -GFAP or p75^NTR^ in limb muscles of ALS and controls.

ALS Donor	NF-L	SYP	S100B	GFAP	p75^NTR^
Da	10% (N = 10)	56% (N = 9)	8% (N = 13)	90% (N = 10)	27% (N = 11)
D1	7% (N = 15)	0% (N = 15)	9% (N = 11)	100% (N = 6)	19% (16)
D3^a^	33% (N = 6)	18% (N = 11)	0% (N = 14)	100% (N = 8)	33% (N = 12)
	54% (N = 28)	50% (N = 24)	16% (N = 25)	100% (N = 22)	82% (N = 17)
D5	33% (N = 18)	24% (N = 17)	33% (N = 6)	100% (N = 7)	57% (N = 7)
D7	0% (N = 2)	0% (N = 6)	–	–	–
Mean±SE	23%±20.6* (N = 79)	25%±23.9* (N = 82)	13%±12.6* (N = 69)	98%±4.5 (N = 53)	44%±25.9^†^ (N = 63)
**Controls**					
Adult (n = 5)	81%±5.7 (N = 76)	95%±4.4 (N = 88)	94%±7.4 (N = 75)	100%±0 (N = 57)	91%±9.6 (N = 55)
Elderly (n = 5)	69%±13.9 (N = 73)	64%±15.2** (N = 127)	55%±10.3** (N = 103)	91%±6.0 (N = 53)	92%±9.7 (N = 46)

a Two separate muscle samples from biceps brachii were examined from donor 3.

* Denotes statistically significant difference between both ALS donors and adult controls and between ALS donors and elderly controls.

** Denotes statistically significant difference between adult control and elderly control (p<0.05).

N: the number of NMJs examined; n: the number of control subjects studied; SYP: synaptophysin.

## Results

### Neuromuscular Junctions

In the present study, EOMs and limb muscles from both adult and elderly controls were included in order to distinguish between the impact of the disease itself and the impact of aging, given that 6 out of the 7 ALS donors were over 60 years of age. In EOMs there was no statistically significant difference in the percentage of NMJs labeled with antibodies against NF-L, synaptophysin, S100B, GFAP and p75^NTR^ between adult and elderly controls (Table 2). +In contrast, in limb muscles there was a statistically significant difference in the percentage of NMJs labeled with antibodies against synaptophysin and S100B between adult and elderly controls (Table 3). Nevertheless, in the limb muscles the difference in the percentage of NMJs labeled with these two antibodies was statistically significant both when comparing ALS donors with adult (synaptophysin, p = 0.0004; S100B, p<0.0001) and ALS donors with elderly (synaptophysin, p = 0.0107; S100B, p = 0.0005) controls.

#### EOMs

**Figure 1:**
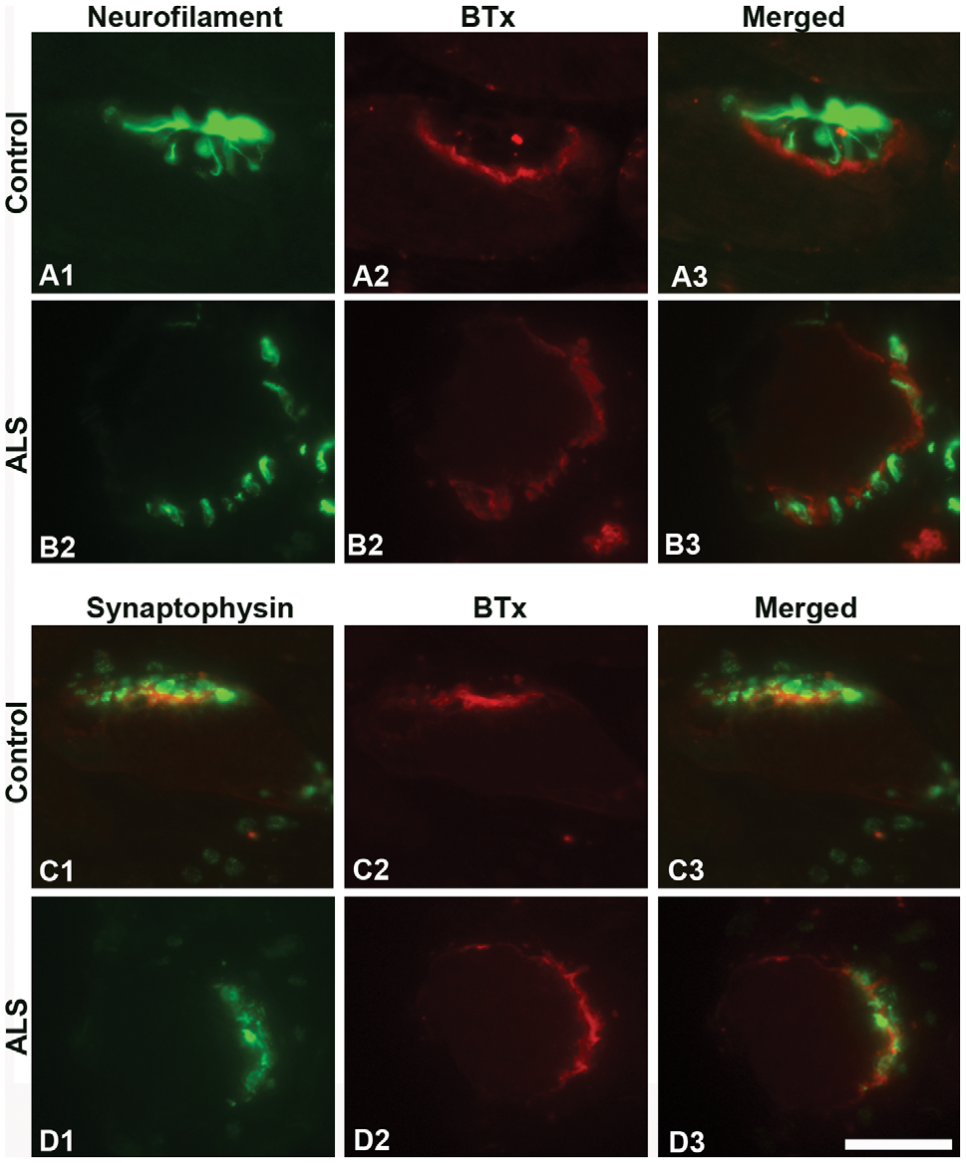
NF-L and synaptophysin at NMJs of EOMs. Light microscopic images of NMJs from adult controls (A and C) and ALS donors (B and D) double-labeled with α-bungarotoxin (BTx, red) and antibodies against NF-L (green; A1, A3, B1, and B3) or synaptophysin (green; C1, C3, D1 and D3). Note the similar staining patterns between controls and ALS donors. Bar = 20 µm.

The majority of NMJs in the EOMs of control and ALS donors were strongly labeled with anti-NF-L (Table 2; Figure 1A, B). In EOMs from both controls and ALS donors, axons visualized by NF-L staining showed contact with the muscle fibers through separate outspread branches in the synaptic area furling into a knot (i.e. the terminal nerve axons; Figure 1A), or were interwoven with postsynaptic labeling by α-BTx (Figure 1B). Anti-synaptophysin labeled the neuromuscular junctions similarly in the EOMs of both controls (adult 88%, elderly 84%) and ALS donors (85%) (Figure 1C, D). Synaptophysin immunolabeling was found in very close vicinity to labeling by α-BTx (Figure 1C3, D3). Intense immunoreactivity with anti-synaptophysin was found in the majority of NMJs, yet moderate to weak immunostaining was also encountered in ALS and control NMJs. No significant differences in the percentage of NMJs labeled with anti-NF-L and anti-synaptophysin were observed between ALS donors and controls. Therefore, nerve-muscle contacts were preserved in the NMJs of the EOMs of ALS donors.

**Figure 2:**
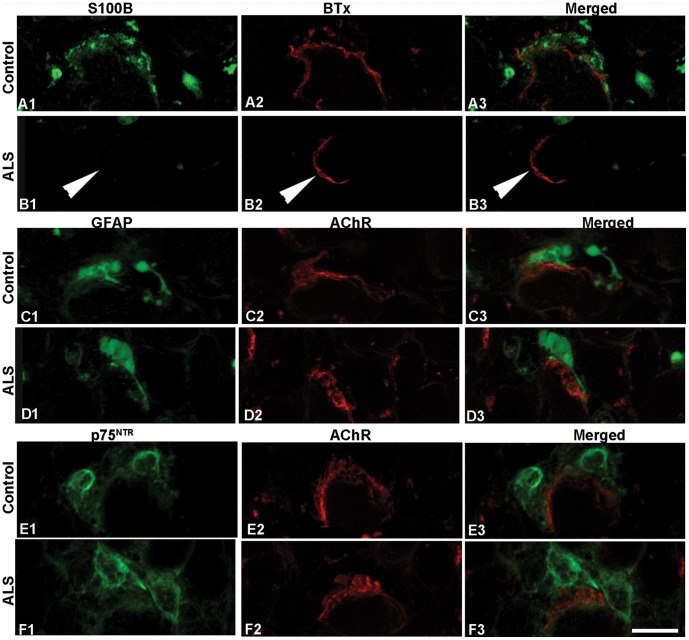
S100B, GFAP and p75^NTR^ at NMJs of EOMs. Confocal images of NMJs from adult controls (A, C, and E) and ALS donors (B, D and F) double-labeled with antibodies (green) against S100B (A, B), GFAP (C, D) and p75^NTR^ (E, F) and α-bungarotoxin (BTx, red in A and B) or antibody against AChR (red in C–F). Note the similar staining patterns between controls and ALS donors with all antibodies, with the exception of S100B. Arrowheads in B denote lack of staining. Bar = 24 µm.

S100B immunoreactivity was generally strong and present in the majority of NMJs from both adult (78%) and old controls (82%; Table 2; Figure 2A; Figure S1). However, S100B labeling was lacking in the vast majority of NMJs in EOMs from ALS (Figure 2B). For instance, in the rectus medialis from donor 7, only 6 NMJs were labeled with anti-S100B out of a total of 96 (6%) NMJs identified with α-BTx (Table 2). The number of NMJs labeled with anti-S100B varied considerably between different donors and also between EOMs from the same donor, ranging from 6% to 69%. On average, 25% of NMJs in EOMs with ALS were labeled, and a statistically significant difference in the percentage of NMJs positive to anti-S100B was observed between ALS donors and controls. Notably, the rectus superior from ALS donor 1, who displayed the highest percentage of NMJs stained with anti-S100B, showed exceptionally high levels of S100B immunoreactivity in nerve bundles.

To determine whether the absence of S100B protein reflected degeneration/absence of terminal Schwann cells in EOMs with ALS, we investigated the distribution of two additional Schwann cell markers, GFAP and p75^NTR^. Strong immunoreactivity for both GFAP and p75^NTR^ was detected adjacent to motor endplates in both ALS and control EOMs (Figure 2C–F; Figure S1). There was close spatial correspondence of all three Schwann cell markers at the NMJ (not shown). In general, the staining with anti-GFAP was mostly found in close apposition and/or partially overlapping with anti-AChR or α-BTx labeling in control EOMs (Figure 2C, D; Figure S1). There were no significant differences in the number of NMJs labeled with anti-GFAP, the labeling intensity and the distribution of anti-GFAP staining observed between ALS donors and the adult and elderly control groups (Table 2; Figure 2C, D). The vast majority of NMJs were labeled with anti-p75^NTR^ in both controls and in ALS donors (Table 2; Figure 2E, F).

#### Limb muscles

**Figure 3:**
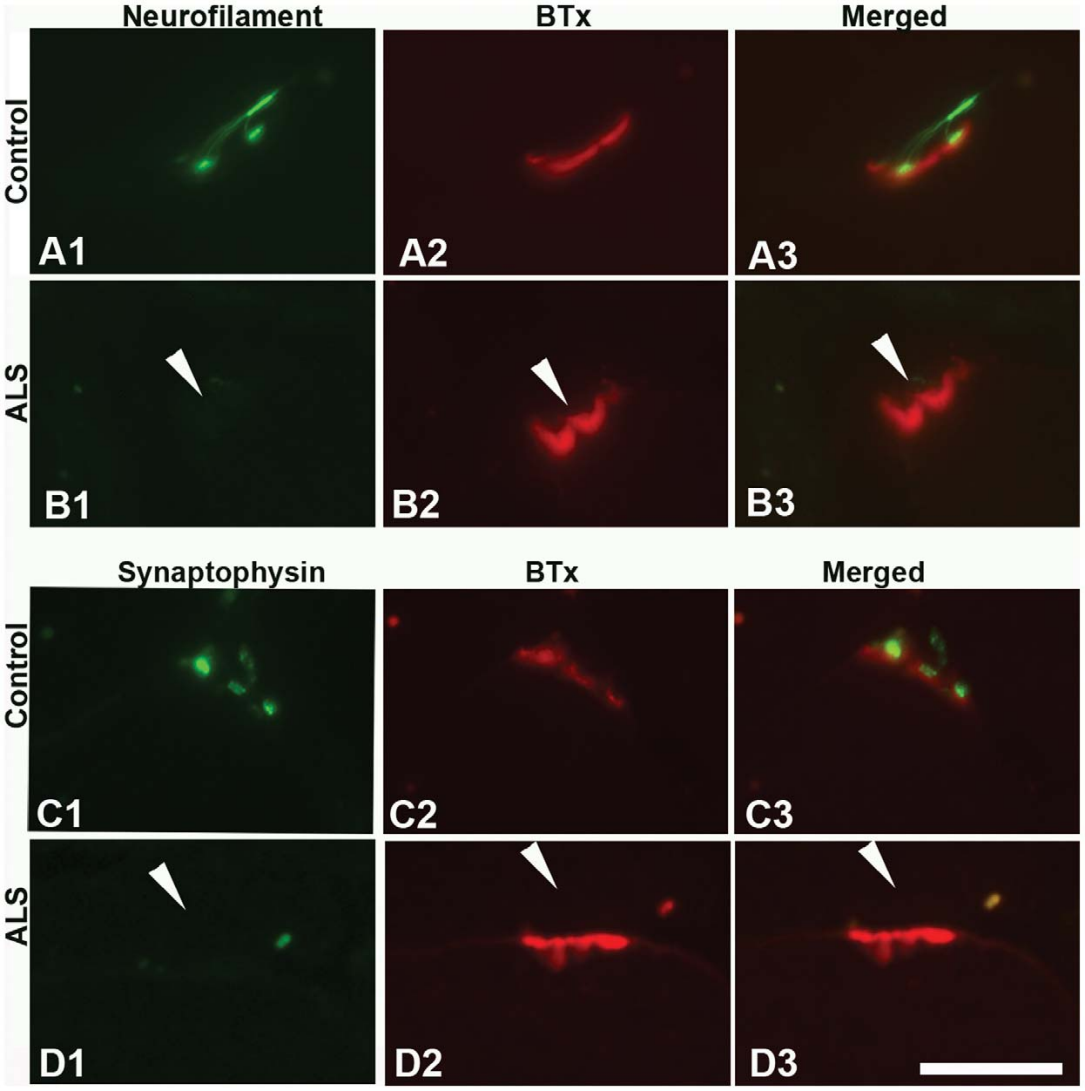
NF-L and synaptophysin at NMJs of limb muscles. Light microscopic images of NMJs of biceps brachii from adult controls (A, C) and ALS donors (B, D) double-labeled with α-bungarotoxin (BTx, red) and antibodies (green) against NF-L (A, B), synaptophysin (C, D). Note the absent staining at NMJs in ALS donors (arrowheads). Bar = 20 µm.

Strong labeling with antibodies against NF-L and synaptophysin was present in the close vicinity of most of the motor endplates labeled by α-BTx in limb muscles of both adult and elderly controls (Table 3; Figure 3A, C). Although adult and elderly control muscles showed similar staining pattern and intensity with these two antibodies, the percentage of NMJs labeled with anti-synaptophysin was significantly decreased in muscle samples from elderly as compared to adult subjects (Table 3). Nevertheless, the number of NMJs with NF-L protein (Figure 3B) and the number of NMJs with synaptophysin (Figure 3D) were significantly decreased in ALS limb muscles as compared to controls (Table 3), indicating significant loss of muscle-nerve contacts in ALS.

**Figure 4:**
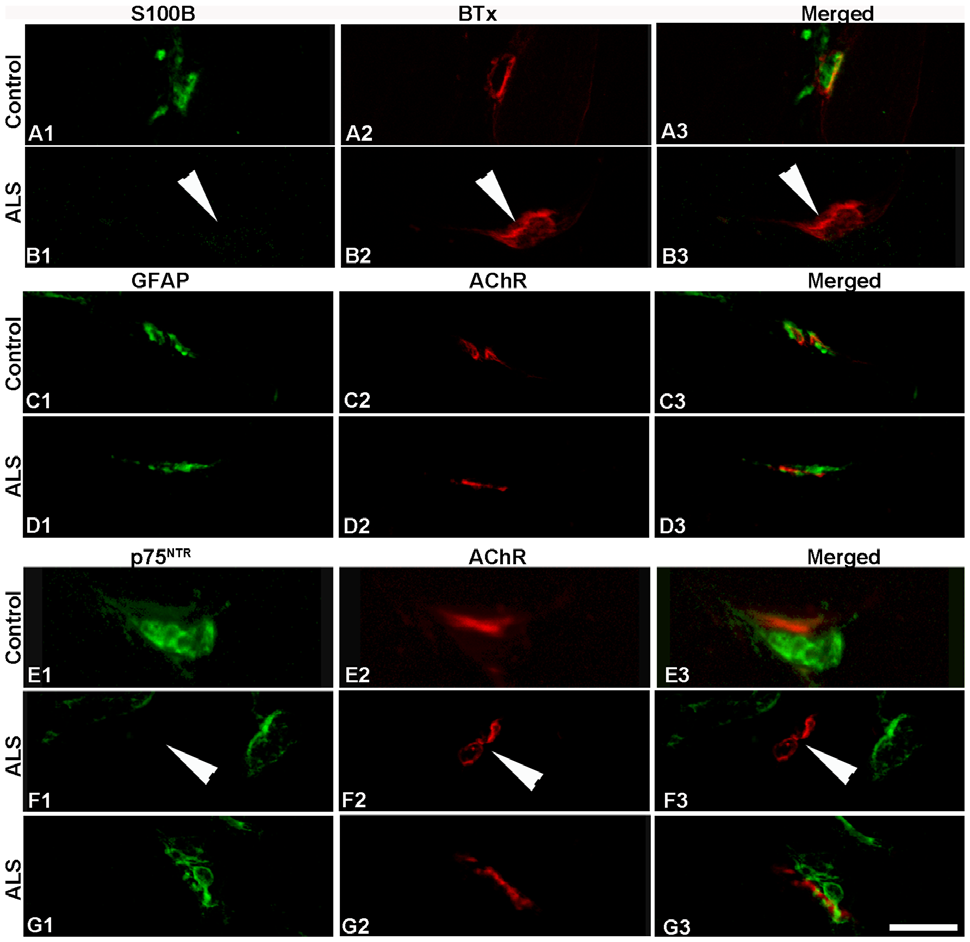
S100B, GFAP and p75^NTR^ at NMJs of limb muscles. Confocal images of NMJs of biceps brachii from adult controls (A, C, and E) and ALS donors (B, D, F and G) double-labeled with antibodies (green) against S100B (A, B), GFAP (C, D) and p75^NTR^ (E, F and G) and α-bungarotoxin (BTx, red in A and B) or antibody against AChR (red in C–G). Note the absent staining at NMJs in ALS donors (arrowheads). Bar = 24 µm.

S100B labeling was mainly present on the nerve side of the NMJs in normal limb muscles (Figure 4A; Figure S2). On average, 94% of NMJs were labeled with anti-S100B in limb muscles from adult controls whereas significant less NMJs (55%) were labeled in elderly controls (Table 3). In ALS limb samples, the vast majority of NMJs (87%) showed no immunoreactivity with anti-S100B (Figure 4B; Figure S2). The population of positive NMJs was significantly decreased as compared to the controls of both age-groups (Table 3).

Virtually all NMJs in limb muscles were strongly labeled with anti-GFAP in both control groups and ALS donors (Table 3; Figure 4C, D). The majority of NMJs in adult (91%) and elderly (92%) control limb muscles were moderately to weakly labeled with antibody against p75^NTR^ (Figure 4E). Significant difference in the percentage of NMJs stained with anti-p75^NTR^ was observed in limb muscles between ALS donors and controls (Table 3). Furthermore, the number of NMJs labeled with anti-p75^NTR^ varied considerably between donors. The donor with the largest population was D3, who had 82% of p75^NTR^-positive NMJs (Table 3; Figure 4G), whereas D1 and Da had only 19% and 27% of NMJs labeled with anti-p75^NTR^, respectively (Figure 4F; Table 3). Regenerating muscle fibers, identified as fibers of very small diameter labeled with the antibody against embryonic myosin heavy chain isoform, were strongly labeled with anti-p75^NTR^ as well as anti-GFAP (not shown).

#### EOMs and limb muscles from SOD1^G93A^ mice

**Figure 5:**
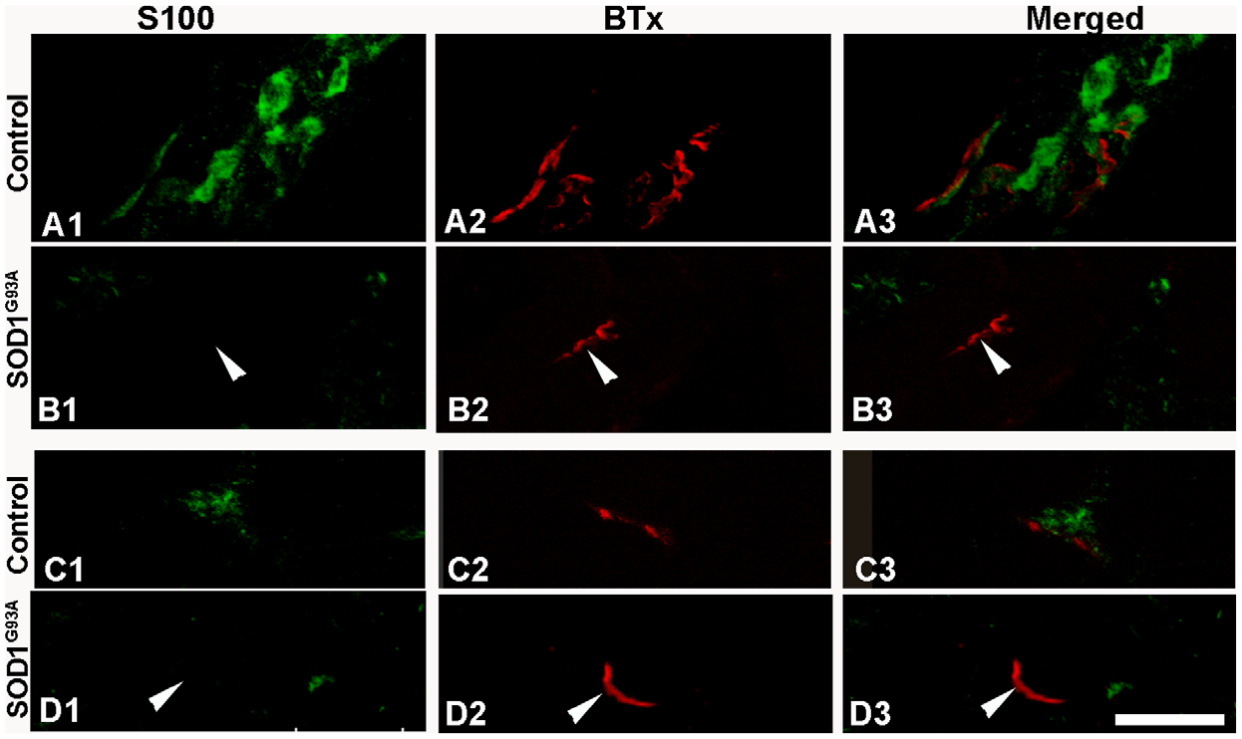
S100 at NMJs of EOM and limb muscles from ALS SOD1^G93A^ mice. Confocal images of NMJs from control (A, C) and SOD1^G93A^ transgenic mice (B, D) at 159 and 157 days, respectively, double-labeled with α-bungarotoxin (BTx, red) and antibody against S100 (green). Note that S100 immunoreactivity was lacking at nerve terminals and motor end plates from both EOMs and limb muscles of transgenic mice at terminal stages. Bar = 24 µm.

Significant decrease in anti-S100 immunoreactivity at NMJs in both EOMs and limb muscles of ALS donors were confirmed on muscles from SOD1^G93A^ mice at terminal stage (Figure 5B, 5D). In contrast, immunoreactivity with anti-S100 was maintained in the majority of NMJs in age-matched control mice (Figure 5A, 5C). The results in SOD1^G93A^ mice were thus similar to those of ALS donors.

### Intramuscular Nerve Bundles

#### EOMs

**Figure 6:**
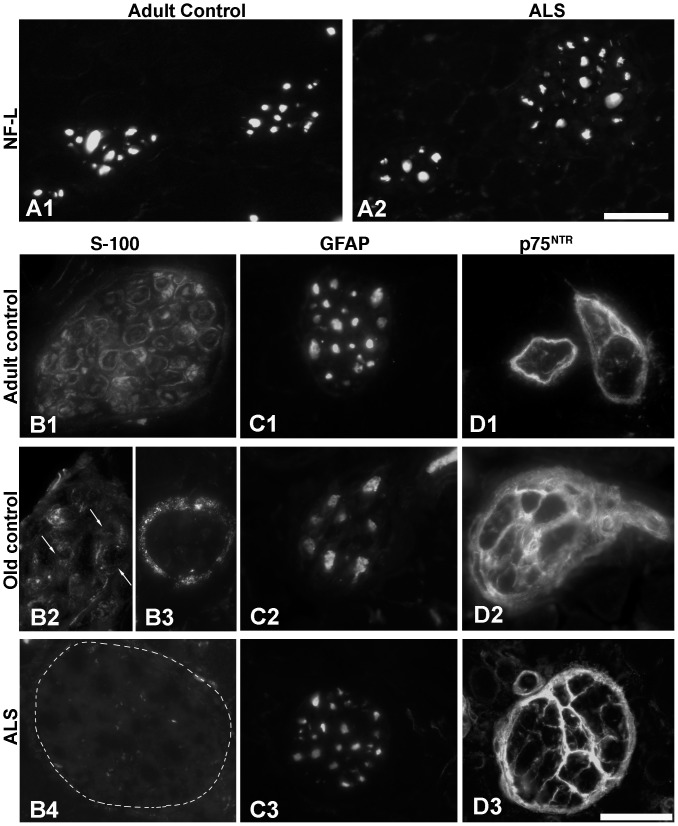
Intramuscular nerve axons in EOMs. Light microscopic images of nerve axons from adult and elderly controls and from ALS donors treated with antibodies against NF-L (A), S100B (B), GFAP (C) and p75^NTR^ (D). Note the absence of S100B in the nerve axons (B3, outlined area) in the ALS donor. Bars = 40 µm.

The antibody against NF-L labeled axons strongly and solidly in EOMs from both young and old controls as well as from ALS donors (Figure 6A1, A2). In nerve bundles of adult control EOMs, anti-S100B immunoreactivity was observed in Schwann cells surrounding each individual axon, and some of the axons inside the nerves were weakly labeled whereas the remaining were unlabeled (Figure 6B1). In old control EOMs the staining intensity with anti-S100B was lower in some nerve bundles, i.e. some Schwann cells were only partially stained or unstained (Figure 6B2) whereas in other nerve bundles S100B labeling was absent from Schwann cells but present discontinuously around the perineurium (Figure 6B3). S100B immunoreactivity was in general not found in Schwann cells around axons nor around perineurium in EOMs of ALS donors (Figure 6B4). Anti-S100B labeling of nerve bundles was lacking in 15 out of 16 muscle specimens from 5 ALS donors, except for low intensity and unevenly distributed staining occasionally seen in some nerve fibers in a few of these specimens (data not shown). The antibody against GFAP labeled axons of nerve bundles strongly and evenly, in both normal and ALS EOMs (Figure 6C1–C3). Strong p75^NTR^ labeling was observed in the perineurium of nerve bundles of young adult controls whereas less intense staining was frequently seen inside the nerves (Figure 6D1). In addition, p75^NTR^ staining which divided the nerve bundle into several “compartments” was occasionally encountered in young control EOMs and more often in old controls (Figure 6D2). In ALS EOMs anti-p75^NTR^ generally delineated the nerve bundles strongly and further divided the nerves into “compartments” (Figure 6D3).

#### Limb muscles

**Figure 7:**
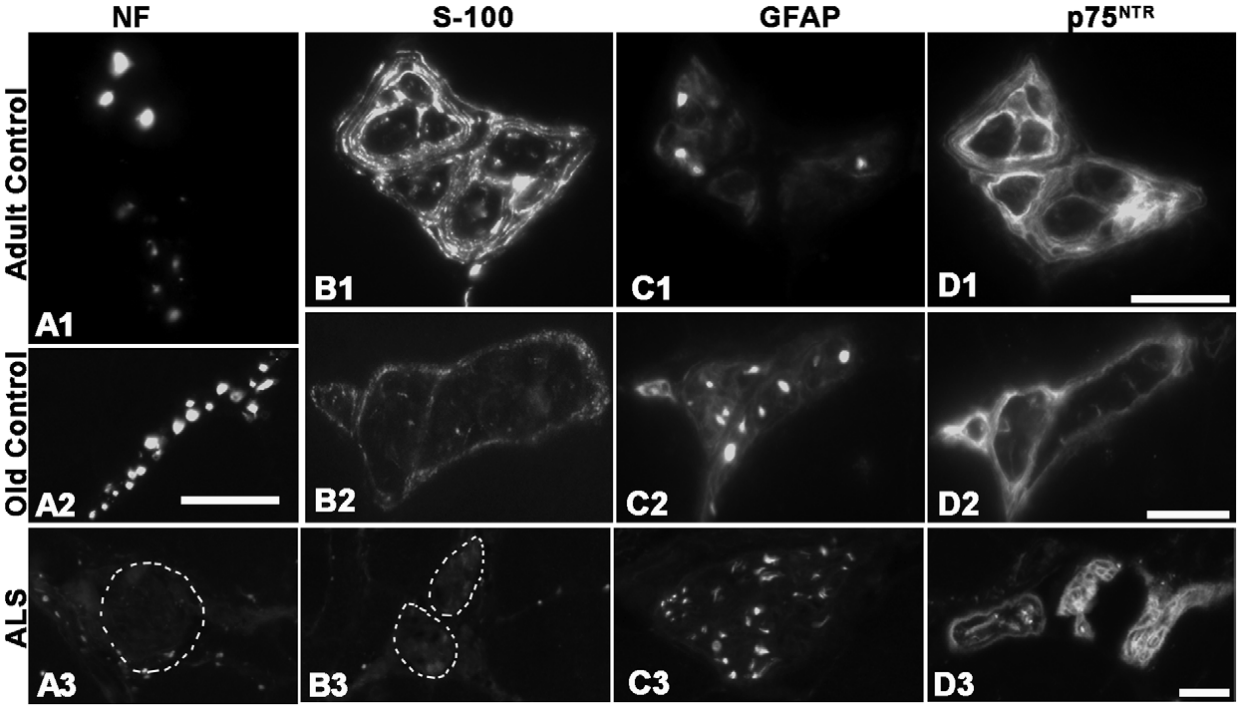
Intramuscular nerve axons in limb muscles. Light microscopic images of nerve axons from adult (top panel) and elderly (middle panel) controls and from ALS donors (bottom panel) labeled with antibodies against NF-L (A), S100B (B), GFAP (C) and p75^NTR^ (D). Note the absence of NF-L (A3) and S100B (B3) in the nerves (outlined areas) of the ALS donors. Bars = 20 µm.

The antibody against NF-L labeled nerve axons strongly in both adult (Figure 7A1) and elderly (Figure 7A2) controls. In contrast, the nerve axons were in general completely unlabeled in ALS limb muscles (Figure 7A3), with the exception of a few nerve bundles from donors 1 and 2 which showed labeling only in sporadic axons (not shown). As in the EOM controls, anti-S100B strongly labeled the Schwann cells around axons and, to some extent, also the axons inside the nerves of adult controls (Figure 7B1), whilst the anti-S100B antibody showed less intense labeling in nerves from elderly (Figure 7B2). Although weak to moderate stainings were occasionally found discontinuously along the perineurium in two donors (not shown), the nerves were mostly unlabeled with anti-S100B in ALS limb muscles (Figure 7B3). Anti-GFAP strongly labeled nerve axons in limb muscles of the control groups (Figure 7C1, C2), and comparable staining patterns were observed in nerve axons of ALS donors (Figure 7C3). Anti-p75^NTR^ strongly labeled the perineurium of nerve bundles in control (Figure 7D1, D2) and ALS limb samples (Figure 7D3). Although few NMJs in the limb muscles from donors D1 and Da showed p75^NTR^ immunostaining as mentioned earlier, their nerve axons were as strongly labeled as in other cases. The nerves unstained with anti-NF-L or anti-S100B were strongly labeled by anti-p75^NTR^ and anti-GFAP in adjacent cross-sections (not shown).

## Discussion

Data on cellular and molecular changes at NMJs in human ALS are scarce in spite of accumulating evidence that loss of contact at the NMJ is an early event in the pathophysiology of ALS, in the transgenic SOD1^G93A^ animal model [5]. The present study is the first to investigate the cellular components of the NMJs of EOMs in terminal human ALS and to demonstrate important differences between EOMs and limb muscles. The main findings of the present study were: (i) NF protein 70 kD subunit decreased dramatically in both nerve axons and NMJs in limb muscles but not in EOMs with ALS; (ii) synaptophysin decreased significantly at NMJs of ALS limb muscles but not of EOMs; (iii) S100B declined significantly in both limb and EOMs of ALS donors, in nerves as well as at NMJs; (iv) p75^NTR^ decreased only at NMJs in ALS limb muscles but not in their nerves. In short, these findings showed that limb muscle nerves and NMJs were much more affected than those in the EOMs, in end-stage ALS donors. This study also showed important differences on the impact of aging at the NMJs of limb muscle versus EOMs. However, even though the synaptic expression of NF-L and synaptophysin declined significantly in aged limb muscles, aging was not a confounding factor since there was still significant difference in synaptophysin expression between ALS donors and elderly controls. We interpret the lack of NF-L and synaptophysin labeling in limb NMJs of terminal ALS donors as a sign of disease related denervation, with loss of contact between motor axons and muscle cells, as described in the terminal transgenic SOD1^G93A^ mouse model of ALS [5]. In this mouse model, progressive axonal retraction starting at the NMJ in the hind limb, was reported as an early event in the pathophysiology of ALS, present before the animals showed symptoms of the disease [5]. Importantly, the present results showing that such loss of contacts between motor axons and muscle fibers does not occur in the EOMs of terminal stage ALS donors, further strengthens our data observed in the SOD1^G93A^ model [8], indicating that EOMs are capable of maintaining their NMJs in spite of the advance of the disease. We have previously reported that the EOMs of terminal ALS donors show far less signs of pathological involvement than the corresponding limb muscles [6], [7], which is also supported by the present study.

A decline of NF-L mRNA levels in the spinal cord and motor neurons was reported in terminal ALS human cases [29], [30], in and in cell culture models of ALS [30]. Cerebrospinal fluid levels of NF-L determined with ELISA are elevated in ALS patients and correlate inversely with disease duration [31]. Double transgenic mice carrying SOD1^G37R^ mutation and overexpressing NF-L or NF heavy proteins showed less pronounced axonal degeneration/loss in their ventral roots and their lifespan was increased by up to 65% [32], [33]. Thus, NF has been suggested to play a protective role in ALS [32]–[34]. NF proteins are produced in the cell bodies of motor neurons and then transported along axons toward the synaptic terminals. Taken together, the present data on NF and synaptophysin can be interpreted to indicate that the human EOMs succeeded in keeping their neuromuscular contacts until the very end-stage of ALS, in contrast to the limb muscles. However, the present data on the significant decrease of NMJs displaying S100B immunoreactivity indicate that they are somehow affected.

The current study showed absence or dramatic decrease of S100B in nerve axons and NMJs in both EOM and limb muscles with ALS. However, strong immunoreactivity with GFAP and p75^NTR^, which are also Schwann cell makers, was detected in all EOM ALS samples. Thus, the absence of S100B immunoreactivity in the NMJs is likely due to down-regulation of S100B in the terminal Schwann cells rather than reflecting the absence of terminal Schwann cells. The fact that S100B protein was also absent or decreased in the NMJs of EOMs and limb muscles from transgenic SOD1^G93A^ mice at terminal stage, suggests that downregulation of S100B is truely related to ALS. Further studies on the transgenic SOD1^G93A^ mouse model are needed to elucidate the temporal aspects and regulation of this process. Decreased levels of S100B protein were detected in the serum [17] and cerebrospinal fluid [18] of ALS patients over the course of the disease. Since S100B is a non-ubiquitous Ca^2+^-modulated protein with multifaceted intracellular and extracellular regulatory roles [35], [36], the significance of its down-regulation at NMJs in ALS is unclear. Because S100B acts as a Ca^2+^ sensor protein to regulate Ca^2+^ homeostasis within terminal Schwann cells and increased intracellular calcium is regarded as a common denominator in motor neuron injury in ALS [37], a possible speculation is that changes in Ca^2+^ homeostasis might be involved in the breakdown of the nerve-muscle contact at the NMJs in ALS.

In ALS limb NMJs, p75^NTR^ was also decreased, although to different levels and independently of changes in S100B (Table 3). p75^NTR^ was totally undetectable in most limb NMJs in ALS donors whereas intensive immunoreactivity was observed in the neighbouring nerve axons. The persistence of p75^NTR^ in nerve trunks but its absence from NMJs in ALS limb muscles is difficult to explain, but it may be a sign of ongoing axonal retrograde changes starting at the NMJ. p75^NTR^ is a neurotrophin receptor which regulates neuronal survival and differentiation as well as neuronal apoptosis at several levels by binding with all neurotrophins. There is evidence that p75^NTR^ plays a crucial role in myogenin differentiation and muscle repair in vivo via NGF/p75^NTR^ signaling pathway [38]. The strong co-expression of embryonic myosin heavy chain, a developmental isoform, and p75^NTR^ reported here indicates ongoing muscle fiber regeneration in certain areas of the human limb muscles with ALS, whereas no corresponding finding was made in the EOMs.

We showed in the current study a distinctly larger impact of ALS on synaptic proteins located in the motor axons and the terminal Schwann cells in limb muscles, in contrast to only mild changes at NMJs in EOMs. Further studies in transgenic mouse models of ALS are underway to investigate the time line of events at the NMJs, in order to shed more light on the roles of terminal Schwann cells, neurotrophins and their receptors in the differences between the EOMs and limb muscles.

## Supporting Information

Figure S1 **S100B, GFAP and p75^NTR^ at NMJs of EOMs.** Light microscopic images of NMJs from controls and from ALS donors double-labeled with α-bungarotoxin (BTx, red) and antibodies (green) against S100B (A–C), GFAP (D, E) and p75^NTR^ (F, G). A1–A2 (Bar = 60 µm), are lower magnification photographs showing several NMJs (arrows) on longitudinally cut muscle fibers. Notice that the EOMs are richly innervated and small nerves labeled with anti-S100B are seen in between muscle fibers. Arrowheads in C1 and C3 denote lack of staining. Bar = 20 µm (B–G).
(TIF)

Figure S2 **S100B, GFAP and p75^NTR^ at NMJs of limb muscles.** Light microscopic images of NMJs of biceps brachii from controls and ALS donors double-labeled with α-bungarotoxin (BTx, red) and antibodies (green) against S100B (A, B), GFAP (C, D) and p75^NTR^ (E–G). Arrowheads denote absence of staining. Bar = 10 µm.
(TIF)
