# Supplementary figures and images for: Distinct Changes in Synaptic Protein Composition at Neuromuscular Junctions of Extraocular Muscles versus Limb Muscles of ALS Donors
Source: PLoS One. 2013 Feb 26;8(2):e57473. doi: 10.1371/journal.pone.0057473 (PMC3582511; doi:10.1371/journal.pone.0057473)

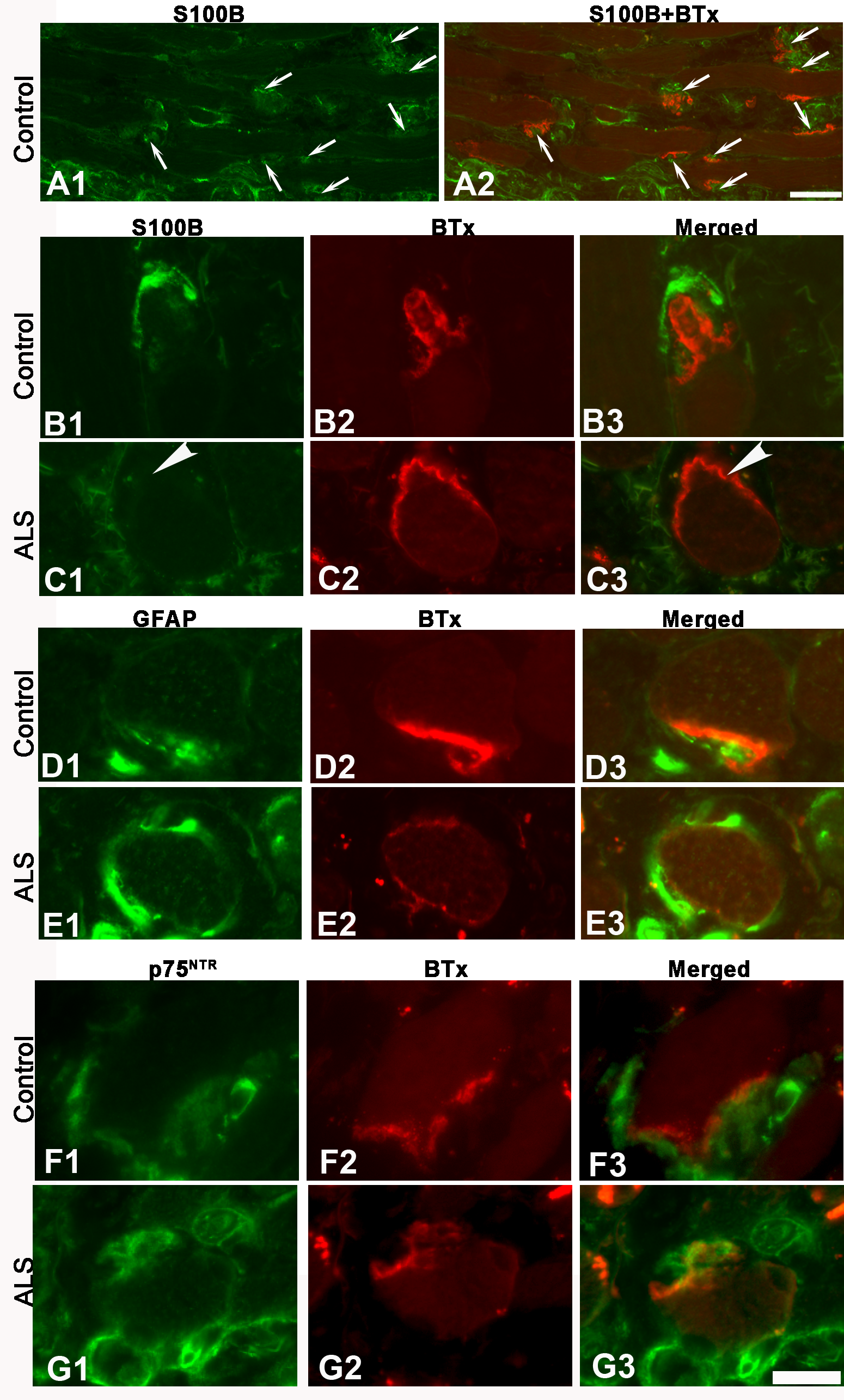

Supplement: Figure S1 — S100B, GFAP and p75NTR at NMJs of EOMs. Light microscopic images of NMJs from controls and from ALS donors double-labeled with α-bungarotoxin (BTx, red) and antibodies (green) against S100B (A–C), GFAP (D, E) and p75NTR (F, G). A1–A2 (Bar = 60 µm), are lower magnification photographs showing several NMJs (arrows) on longitudinally cut muscle fibers. Notice that the EOMs are richly innervated and small nerves labeled with anti-S100B are seen in between muscle fibers. Arrowheads in C1 and C3 denote lack of staining. Bar = 20 µm (B–G). (TIF) [file pone.0057473.s001.tif]

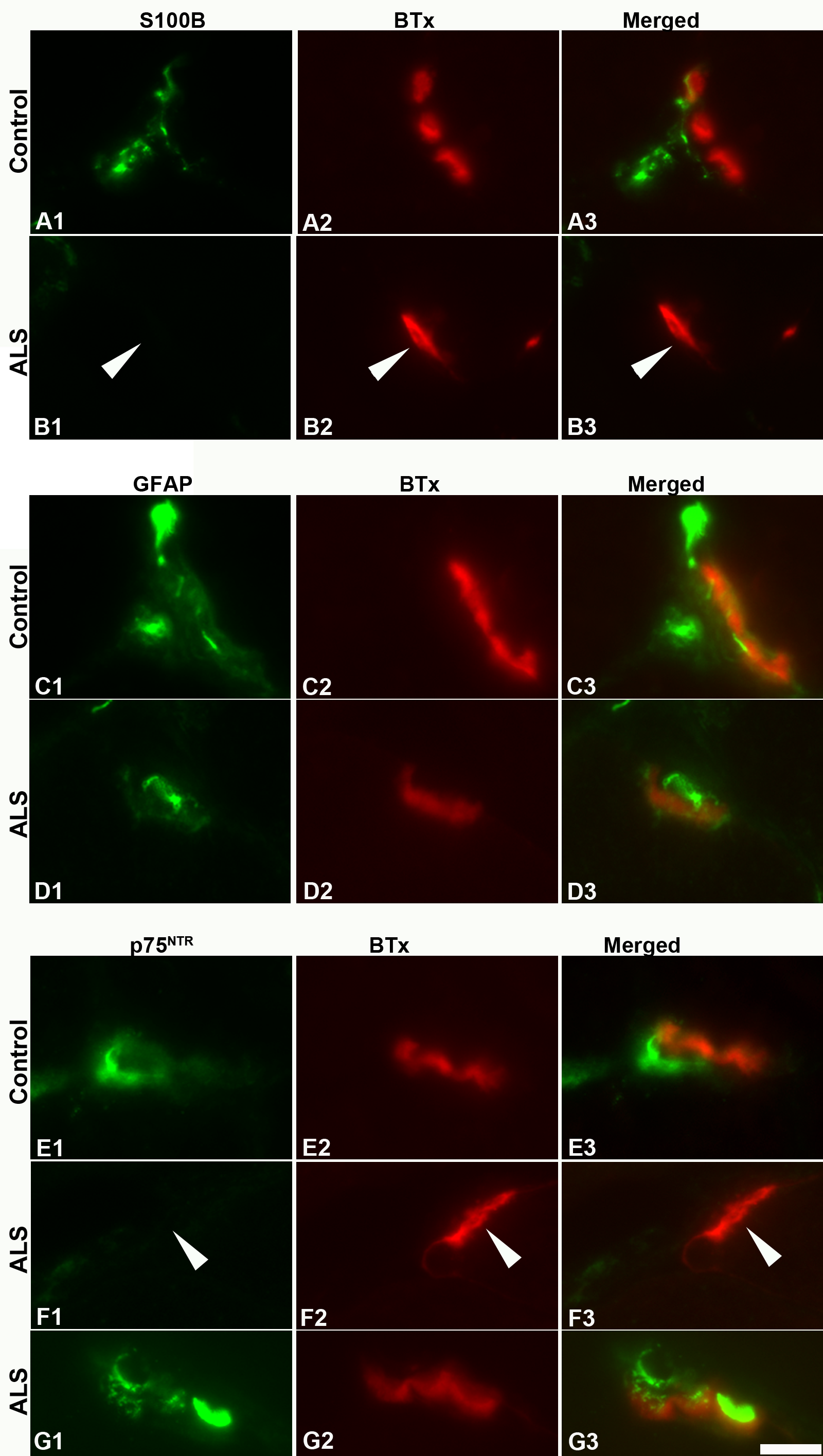

Supplement: Figure S2 — S100B, GFAP and p75NTR at NMJs of limb muscles. Light microscopic images of NMJs of biceps brachii from controls and ALS donors double-labeled with α-bungarotoxin (BTx, red) and antibodies (green) against S100B (A, B), GFAP (C, D) and p75NTR (E–G). Arrowheads denote absence of staining. Bar = 10 µm. (TIF) [file pone.0057473.s002.tif]
